# Maternal drug use and the risk of anorectal malformations: systematic review and meta-analysis

**DOI:** 10.1186/s13023-018-0789-3

**Published:** 2018-05-10

**Authors:** Nadine Zwink, Ekkehart Jenetzky

**Affiliations:** 1 Department of Child and Adolescent Psychiatry, University Medical Center of the Johannes Gutenberg University, Mainz, Germany; 2 Child Center Maulbronn GmbH, Hospital for Pediatric Neurology and Social Pediatrics, Maulbronn, Germany

**Keywords:** Anorectal malformations, Imperforate anus, Anal atresia, Birth defects, Risk factors, Medication, Drug intake, Pregnancy

## Abstract

**Background:**

Origin of anorectal malformations (ARM) are considered multifactorial. Several genetic and non-genetic risk factors are discussed in literature. Maternal periconceptional medical drug use as possible risk factor, however, has not been reviewed systematically.

**Methods:**

Studies published between 1977 and April 2017 were reviewed through systematic search in PubMed, ISI Web of Knowledge and Scopus databases. Furthermore, related and cross-referencing publications were reviewed. Pooled odds ratios (95% confidence intervals) were determined to quantify associations of maternal periconceptional use of folic acid, multivitamins, anti-asthma medication (separated in any anti-asthma medication, inhaled corticosteroids and salbutamol), thyroid hormone supplements, psychiatric drugs (separated in antidepressants, any selective serotonin reuptake inhibitors [SSRI], sertraline, citalopram, fluoxetine, paroxetine, hypnotics and benzodiazepine) and aspirin with ARM using meta-analyses.

**Results:**

Thirty-seven studies that reported on the association between maternal periconceptional drug intake and infants born with ARM were included in this review. These were conducted in the United States of America (*n* = 14), Sweden (*n* = 6), Hungary (*n* = 5), Germany (*n* = 3), the Netherlands (*n* = 3), Denmark (*n* = 2), France (*n* = 2), Norway (*n* = 1) and the UK (*n* = 1). However, only few of these studies reported on the same risk factors. Studies were heterogeneous with respect to case numbers, period ingestion of medical drug use, control selection and adjustment for covariates. Consistently increased risks were observed for any anti-asthma medication, and hypnotics and benzodiazepine, but not for folic acid, multivitamins, inhaled corticosteroids, salbutamol, thyroid hormone supplements, antidepressants, any SSRI, sertraline, citalopram, fluoxetine, paroxetine and aspirin. In meta-analyses, pooled odds ratios (95% confidence intervals) for any anti-asthma medication, and hypnotics and benzodiazepine were 1.64 (1.22–2.21), and 2.43 (1.03–5.73), respectively.

**Conclusion:**

Evidence on maternal drug use before conception and during pregnancy as risk factor for ARM from epidemiological studies is still very limited. Nevertheless, the few available studies indicate any anti-asthma medication, and hypnotics and benzodiazepine to be associated with increased risks. Further, ideally large-scale multicenter and register-based studies are needed to clarify the role of maternal drug intake for the development of ARM.

## Background

Anorectal malformations (ARM) are rare birth defects concerning anus and rectum. Approximately 1 in 2500 to 1 in 5000 new born babies are affected [1–3]. Different degrees of severity are distinguished, ranging from mild anal stenosis over anal atresia with or without fistula to persistent cloaca or even cloacal exstrophy [4]. Furthermore, approximately 64% of all ARM patients have one or more additional extra-anal anomalies [5]. ARM affect several socioeconomic and ethnic groups [6–10]. Boys seem to be at a slightly higher risk than girls (1.3:1) [11]. It is assumed that the defects occur during the 4th to 8th week of fetal development [12–17]. Knowledge about the causes, however, is still sparse. There are assumptions that genetic factors encourage the development of ARM [18–25], but no single gene or chromosomal locus has been identified so far as the cause of all or even of a majority of ARM. In recent years, several potential non-genetic risk factors for ARM were assessed, with often contrary results, among them prenatal exposures of the parents to lifestyle factors (tobacco, alcohol, caffeine, illicit drugs) and occupational hazards [26], to chronic diseases [27, 28], fever [27, 29] and injuries [30]. Maternal overweight, obesity and diabetes, however, indicate to be associated with increased risks for ARM [26]. In addition, a relationship between a single umbilical artery and ARM is suspected [31]. Assisted reproductive techniques also pose a strongly increased risk for ARM [32–38], but it remains unclear whether the procedure itself or underlying parental infertility cause the defects. Besides these non-genetic factors, the influence of maternal drug intake before and during pregnancy, such as vitamin A overdose [39] or deficiency [40], multivitamin [27], folic acid [27, 41], anti-asthmatic drugs [42] or benzodiazepine lorazepam [43], is subject to ongoing debate.

We conducted a systematic review and meta-analysis of epidemiological studies to summarize current evidence on the relationship between maternal drug intake and ARM, and to identify knowledge deficits that need to be addressed in future research.

## Methods

### Identification of studies and study selection

A literature search was carried out to identify epidemiological studies assessing the association between maternal medical drug intake before conception and during the first trimester of pregnancy and anorectal malformations. Relevant studies published in English were systematically searched in PubMed, ISI Web of Knowledge and Scopus databases by using various combinations of the following terms: (congenital malformation(s), congenital abnormality, congenital abnormalities, birth defect(s), anorectal malformation(s), anorectal atresia, anal atresia, imperforate anus) AND (medical drug(s), drugs(s), medication, medicament, medicine, pharmaceutical, dietary supplements, folic acid, (multi-)vitamins, vitamin A, vitamin B, vitamin C, iron). Duplicate articles were deleted. Each title and abstract was checked for relevance. The full text was reviewed if the abstract indicated that the article reported an association between ARM and maternal use of medical drugs. Furthermore, the identified articles were reviewed for related articles and cross-referring publications.

### Inclusion criteria

Articles were included if they reported on associations of anorectal malformations with maternal medical drug intake. When available, data of ARM infants with isolated anomalies (no additional major defects) were preferred to data of ARM infants with multiple defects. Articles were excluded if the reported number of ARM cases was less than two. ARM infants analyzed only in a group with other anomalies like intestinal or tracheo-esophageal atresias were also excluded because of concern that associations of risk factors with these anomalies might be different from associations with ARM. In addition, articles describing medical drug use in animal models were also excluded. Searches were restricted to English-language articles.

### Data extraction

Two reviewers independently assessed the articles and extracted the following key information in a standardized manner: first author, year, country, study design, characteristics of the study population, period of data acquisition, assessed medical drug(s) and the respective measures of odds ratio or risk ratio (see below), as well as covariates adjusted for in the analysis. Initial disagreements on classifications of study characteristics were resolved by discussion within the team of authors. Such disagreements included the presentation of case and control numbers in some studies as well as the presentation of the maternal age at the time of data acquisition. In such a case, the authors came together and discussed the topics. As one result, footnotes were included in Table 1 to offer the opportunity to present case and control numbers even more accurately.

**Table 1:** Case-control studies reporting on the association of ARM and the maternal use of medical drugs

			Study population					
			No. participants					
Ref.	First author, year	Country	Cases	Controls	Age range	Setting, control type	Data acquisition (period)	Assessed medical drug(s)
[28]	Zwink, 2016	Germany	158	474^a^	< 18 - ≥30	population-based, no major birth defects	Data from the German Network for congenital uro-rectal malformations and Malformation Monitoring Centre Saxony-Anhalt of the Otto-von-Guericke University in Magdeburg (2009–2011)	Folic acid, multivitamins
[52]	Furu, 2015	Norway	799	2,303,647	≤24 - ≥45	population-based, all births	Data from nationwide Nordic health registers (Denmark, Finland, Iceland, Norway, Sweden) (1996–2010)	Antidepressants (any SSRI, citalopram, sertraline)
[73]	Garne, 2015	Denmark	772 265^c^	53,402	< 25 - ≥40	population-based, non-chromosomal and chromosol anomalies	Data from the EUROmediCAT registries (1995–2010)	Anti-asthma medication (any asthma medication, inhaled ß_2_-agonists, inhaled corticosteroids)
[50]	Wemakor, 2015	UK	392	2,177,977	N.A.	registry-based, other congenital malformations	Data from 12 EUROCAT congenital anomalies registries (1995–2009)	Antidepressants (any SSRI, fluoxetine, paroxetine, citalopram, sertraline, escitalopram)
[53]	Zwink and Choinitzki, 2015	Germany	123	140	< 18 - ≥30	population-based, other congenital malformations^a^	Data from the German Network for congenital uro-rectal malformations (2009–2012) and Consortium of Genetic Risk for Esophageal Atresia (2011–2012)	Anti-asthma medication, thyroid hormone supplementations, iron intake, folic acid, multivitamins
[57]	Gilboa, 2014	USA	176	4525	< 20 - ≥35	population-based, all births	Data from the National Birth Defects Prevention Study (1997–2005)	Vitamin E
[75]	Källén and Wikner, 2014	Sweden	588	48,012	< 20 - ≥45	population-based, all births	Data from the nationwide Swedish Medical Birth Register (1996–2011)	Thyroxin
[41]	Wijers, 2014	The Netherlands	643^b^	714	< 18 - ≥30	hospital-based, no major birth defects	Questionnaire (1990–2012)	Folic acid
[76]	Källén, 2013	Sweden	590	69,749	< 20 - ≥45	population-based, all births	Data from the nationwide Swedish Medical Birth Register (1996–2011)	Opioids, anticonvulsants, neuroleptics other than dixyrazine or prochloperazine, dixyrazine or prochlorperazine, sedatives or hypnotics, antidepressants
[49]	Pasternak, 2013	Denmark	52	1,222,503	N.A.	register-based, all births	Data from the nationwide administrative and health care registries in Denmark (1997–2011)	Metoclopramide
[54]	Polen, 2013	USA	741	8002	< 30 - ≥30	population-based, no birth defects	Data from the National Birth Defects Prevention Study (1997–2007)	Antidepressants, venlafaxine
[67]	Yau, 2013	USA	274	7606	< 25 - ≥34	population-based, no birth defects	Data from the Slone Epidemiology Center Birth Defects Study (1993–2010)	Decongestants (pseudoephedrine)
[59]	Lin, 2012	USA	285	6726	< 20 - ≥35	population-based, no birth defects	Data from the National Birth Defects Prevention Study (1997–2003)	Anti-asthma medication (bronchodilator use), anti-inflammatory use
[58]	Hernandez, 2012	USA	540	5546	< 20 - ≥35	population-based, no birth defects	Data from the National Birth Defects Prevention Study (1997–2004)	Non-steroidal anti-inflammatory drugs (aspirin, ibuprofen, naproxen)
[60]	van Gelder, 2011	The Netherlands	16	65,287	< 20 - ≥40	population-based, no major birth defects	Data from the Norwegian Mother and Child Cohort Study (1999–2006)	Non-steroidal anti-inflammatory drugs
[55]	Reefhuis, 2011	USA	582	6406	< 25 - ≥40	population-based, no birth defects	Data from the National Birth Defects Prevention Study (1997–2005)	Clomiphene citrate
[56]	Reis and Källén, 2010	Sweden	428	1,062,190	< 20 - ≥45	population-based, all births	Data from the nationwide Swedish Medical Birth Register (1995–2007)	Antidepressants
[27]	van Rooij, 2010	The Netherlands	85	650	≥35	hospital-based, no major birth defects	Questionnaire (cases 1996–2008, controls 1996–2004)	Folic acid, multivitamins
[61]	Browne, 2009	USA	534	5875	12 - ≥35	population-based, no birth defects	Data from the National Birth Defects Prevention Study (1997–2004)	Anti-thyroid medication
[63]	Crider, 2009	USA	470	5008	< 18–49	population-based, no birth defects	Data from the National Birth Defects Prevention Study (1997–2003)	Antibacterial medication (any antibacterial, penicillins, erythromycins, nitrofurantoins, sulfonamides, cephalosporins)
[62]	Carter, 2008	USA	209	4774	N.A.	population-based, no birth defects	Data from the National Birth Defects Prevention Study (1997–2003)	Antifungal drugs
[68]	Alwan, 2007	USA	418	4092	< 35 - ≥35	population-based, no birth defects	Data from the National Birth Defects Prevention Study (1997–2002)	Antidepressants
[42]	Källén and Otterblad Olausson, 2007	Sweden	495	40,728	N.A.	population-based, all births	Data from the nationwide Swedish Medical Birth Register (1995–2004)	Anti-asthma medication (ß_2_-adrenergic agonists, inhaled corticosteroids, anticholinergic drugs, cromoglicic acid, xanthines, leucotrien receptor antagonists)
[77]	Källén, 2007	Sweden	11	873,383	< 20 - ≥45	population-based, all births	Data from the nationwide Swedish Medical Birth Register (1995–2004)	Folic acid
[64]	Louik, 2007	USA	215	5860	N.A.	population-based, no birth defects	Data from the Slone Epidemiology Center Birth Defects Study (1993–2004)	Antidepressants (any SSRI, fluoxetine, sertraline, paroxetine, citalopram, non-SSRI antidepressant)
[66]	Czeizel, 2004	Hungary	220	38,151	< 25 - > 29	national-based, no birth defects	Data from the Hungarian Congenital Abnormality Registry (1980–1996) and National Birth Registry of the Central Statistical Office (1996–1992)	Folic acid, multivitamins
[69]	Czeizel, 2004	Hungary	220	38,151	< 25 - > 29	national-based, no birth defects	Data from the Hungarian Congenital Abnormality Registry (1980–1996) and National Birth Registry of the Central Statistical Office (1996–1992)	Multivitamins
[6]	Correa, 2003	USA	50	3029	< 20 - ≥30	population-based, no birth defects	Data from the Atlanta Birth Defects Case-Control Study (1968–1980)	Multivitamins
[65]	Czeizel, 2003	Hungary	220	38,151	< 25 - > 29	national-based, no birth defects	Data from the Hungarian Congenital Abnormality Registry (1980–1996) and National Birth Registry of the Central Statistical Office (1996–1992)	Diazepam
[78]	Källén and Mottet, 2003	Sweden	8	504,660	< 19–45	population-based, all births	Data from the nationwide Swedish Medical Birth Register (1995–2001)	Meclozine
[43]	Bonnot, 2001	France	6	13,703	N.A.	population-based, all births	Data from the French Central-East Registry of congenital malformations (1976–1998)	Lorazepam
[70]	Czeizel, 2001	Hungary	220	38,151	< 25 - > 29	national-based, no birth defects	Data from the Hungarian Congenital Abnormality Registry (1980–1996) and National Birth Registry of the Central Statistical Office (1996–1992)	Cephalosporin (cephalexin, cefuroxime)
[51]	Myers, 2001	USA	50	222,264	N.A.	provinces-based, all births	Data from a public health campaign conducted in China (1993–1995)	Folic acid
[71]	Czeizel, 2000	Hungary	220	38,151	< 25 - > 29	national-based, no birth defects	Data from the Hungarian Congenital Abnormality Registry (1980–1996) and National Birth Registry of the Central Statistical Office (1996–1992)	Acetylsalicylic acid
[72]	Stoll, 1997	France	108, 51^**c**^	108	F: mean age 26.9, M: mean age 29.9	hospital-based, no birth defects	Interview (1979–1995)	Antibiotics, antispasmodics, estrogens and other miscellaneous medication
[74]	Angerpointer, 1981	Germany	78 78 78 78	210^d^ 169^e^ 75^f^ 53^g^	< 20 - > 40	hospital-based, other malformed infants	Questionnaire (1970–1974)	Antiemetic, analgetic, laxative and antihypotensive drugs, and iron preparations
[79]	Heinonen, 1977	USA	13	N.A.	N.A.	population-based, no controls	Data from the Collaborative Perinatal Project (1958–1965)	Aspirin

*N.A.* not available, *SSRI* selective serotonin reuptake inhibitors, *EA/TEF* esophageal atresia with or without tracheoesophageal fistula, *ARM* anorectal malformation

^a^Control group included *n* = 98 patients with isolated EA/TEF and *n* = 42 patients with the combined phenotype of EA/TEF and ARM

^b^Included 493 cases from the Netherlands and 150 cases from Germany

^c^ARM infants with isolated (no additional major defects) anomaly

^d^Control group includes 41 infants with esophageal atresia, 41 with stenosis/atresia of the small and large bowel, 75 with Hirschsprung’s disease, 28 with omphalocele and 25 with gastroschisis

^e^Control group includes 41 infants with esophageal atresia, 75 with Hirschsprung’s disease, 28 with omphalocele and 25 with gastroschisis

^f^Control group includes 75 infants with Hirschsprung’s disease

^g^Control group includes 28 infants with omphalocele and 25 with gastroschisis

Associations between maternal medical drug intake and ARM are presented by odds ratios (OR) and their 95% confidence intervals (CI). Alternatively, reported risk ratios (RR) are shown. In one case, only prevalence values were presented. Unadjusted values were recalculated by the Review Manager Software, version 5.3.5 (The German Cochrane Centre, Freiburg, Germany) to validate the results. When measures of associations were not explicitly reported, they were derived from data provided in the articles.

### Meta-analyses

Meta-analyses were performed for risk factors for which results were available from at least three studies. Heterogeneity was assessed by the χ^2^ and I^2^ statistics. When the number of studies is low or when sample sizes are small, the power of the χ^2^ test is low. The I^2^ measure describes the proportion of total variation in effect estimates across studies that is due to heterogeneity rather than sampling error [44]. Fixed and random effects models were calculated by the R© software, version 3.2.4 (The R Foundation for Statistical Computing, Vienna) using standard meta-analysis methods. The fixed effects model was used to estimate the variance of the summary odds ratio when study heterogeneity was low (I^2^ ≤ 25) and the random effects model when study heterogeneity was moderate to high (I^2^ > 25) [45, 46]. Indication of publication bias was assessed by Begg and Mazumdar rank correlation test [47] and Egger’s test [48] (*P* < 0.1).

## Results

### Literature search result

**Fig. 1:**
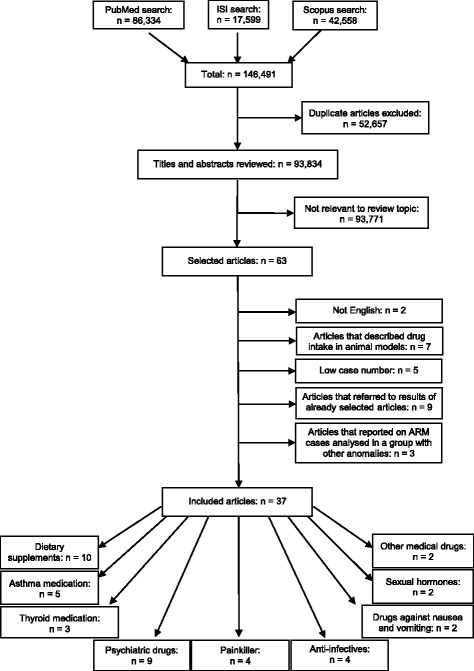
Flow diagram of the literature search process

In total, 146,491 articles were found (Fig. 1). After removal of 52,657 duplicates, 93,834 titles and abstracts were reviewed. Sixty-three articles appeared to be potentially relevant for inclusion in the review. Of these, two articles were excluded because they were published in Spanish or French, seven articles because they described the use of medical drugs in animal models, five articles because of too low case numbers (*n* < 2), nine articles because they referred to results of already selected articles and further three articles because they reported on ARM cases analyzed in a group with other anomalies. Finally, 37 articles were included in the review. Among the included studies, 10 provided data on the association of ARM with maternal periconceptional dietary supplements, including folic acid, iron and (multi-)vitamin use, five to asthma medication, three to thyroid medication, nine to psychiatric drugs, four to painkiller, four to anti-infectives, two to drugs against nausea and vomiting, two to sexual hormones and further two to other medical drugs.

### Studies included in this review

Details on the 37 studies, which were published from 1977 to April 2017, are shown in Table 1. Studies were mainly conducted in the USA (*n* = 14). The remaining studies were conducted in Sweden (*n* = 6), Hungary (*n* = 5), Germany (*n* = 3), the Netherlands (*n* = 3), Denmark (*n* = 2), France (*n* = 2), Norway (*n* = 1) and the UK (*n* = 1). Recruitment was population−/national-based in 30 studies, province-based in one study and hospital-based in four studies. For data acquisition, two studies relied on register-based data [49, 50]. Data acquisition periods varied from 2 years [28, 51] to 22 years [43].

Case numbers ranged from six ARM cases [43] to 799 ARM cases [52]. Children with known chromosomal anomalies were excluded in 20 studies [27, 28, 41, 42, 49, 50, 53–66]. Twenty-one studies used infants with no (major) birth defects as control group [6, 27, 28, 41, 54, 55, 58–72] and four studies used malformed infants with other anomalies than ARM [50, 53, 73, 74]. Controls of the remaining 11 studies were all infants born in the same settings during the respective study period [42, 43, 49, 51, 52, 56, 57, 75–78]. One study only reported on prevalences and did not use a control group [79]. Only nine studies examined the association between maternal medical drug intake and ARM infants with isolated anomalies [41, 53, 57, 59, 65, 69–71, 73].

Most of the studies asked for a period ingestion of medical drug use before and during pregnancy. However, there was no unique definition of the “periconceptional” period of time, varying from 3 months before conception until the end of the third month in pregnancy [6, 28, 53, 61, 66] or through the last month of pregnancy [57], from 2 months before conception though 1 month in pregnancy [33], from 4 weeks before conception until 10 weeks after conception [27, 41] or until 3 months in pregnancy [54, 59, 63, 64, 68], from 2 weeks before conception until 2 weeks after conception [69] or until 4 weeks up to 3 months after conception [65, 71]. Other studies asked there participants for a period ingestion of medical drug use in the first trimester of pregnancy, defined as start of pregnancy until 12 weeks after pregnancy [42, 43, 49, 51, 58, 60, 62, 67, 68, 73, 78] or as the period from the first day of the last menstrual period up to the 12th week of gestation [50, 52], or in general for the use in early pregnancy [56, 75–77] or through the whole pregnancy [70, 74, 79]. Dosage was reported in 12 studies [49, 57, 59, 62, 65–71, 78], exact timing/frequency and/or duration in 16 studies [49, 54, 57–63, 65–67, 69–71, 78]. The exact international World Health Organization’s Anatomical Therapeutic Chemical (ATC) classification [80, 81] was used in seven studies [50, 52, 56, 73, 75, 76, 78]. In addition, nine studies linked all medication to the Slone Drug Dictionary [82], a computerized coding system [54, 57–59, 61–63, 67, 68].

### Findings for the reviewed risk factors

Study results as well as the covariates adjusted for are shown in Tables 2, 3, 4, 5, 6, 7, 8, 9 and 10.

**Table 2:** Associations between ARM and maternal use of dietary supplements

		Maternal use of dietary supplements			
Ref.	First author, year	Exposure	OR_crude_ [95% CI]	OR_adj_ [95% CI]	Adjustment/matching factors
[28]	Zwink, 2016	Folic acid	1.2 [0.54, 2.48]	1.0 [0.29, 3.40]	Adjusted for gender and birth year of the child, maternal age and BMI
		Multivitamins	0.5 [0.11, 2.46]	1.2 [0.21, 7.00]	
[53]	Zwink and Choinitzki, 2015	Folic acid	0.9 [0.49, 1.55]^b^	–	–
			0.8 [0.39, 1.83]^c^	–	
			0.9 [0.57, 1.50]^a,d^	–	
		Iron	0.9 [0.43, 2.05]^b^	–	
			2.4 [0.62, 10.72]^c^	–	
		Multivitamins	1.8 [0.61, 5.60]^b^	–	
			0.5 [0.18, 1.46]^c^	–	
			1.1 [0.48, 2.36]^a,d^	–	
[57]	Gilboa, 2014	Vitamin E	–	1.1 [0.65, 1.74]^g^	Adjusted for study center and maternal total energy intake, folate intake (dietary folate equivalents), race/ethnicity, age, education, pre-pregnancy body mass index, smoking, alcohol use and use of folic acid supplements
				1.7 [1.01, 2.72]^h^	
				1.5 [0.88, 2.51]^i^	
[41]	Wijers, 2014	Folic acid	–	1.1 [0.8, 1.4]^j^	Adjusted for maternal education
			–	1.0 [0.7, 1.5]^k^	
			–	1.1 [0.7, 1.6]^l^	
[27]	van Rooij, 2010	Folic acid	1.0 [0.6, 1.7]	–	–
		Multivitamins	1.6 [1.0, 2.7]	–	
[77]	Källén, 2007	Folic acid	0.9 [0.51, 1.69]^a^	1.0 [0.55, 1.84]	Year of birth, maternal age, parity, maternal smoking, and number of previous miscarriages
[66]	Czeizel, 2004	Folic acid	0.5 [0.17, 1.23]^e^	–	–
			0.4 [0.17, 0.88]^f^	–	
		Multivitamins	0.2 [0.02, 1.69]	–	
[69]	Czeizel, 2004	Multivitamins	0.3 [0.01, 2.35]	–	–
[6]	Correa, 2003	Multivitamins	0.9 [0.45, 1.67]	–	–
[51]	Myers, 2001	Folic acid	0.5 [0.29, 0.88]^m,n^	0.6 [0.33, 1.07]^m,n^	Maternal age
			0.5 [0.24, 1.04]^m,o^	0.7 [0.31, 1.42]^m,o^	
			0.9 [0.28, 2.96]^m,p^	0.9 [0.27, 3.06]^m,p^	
			0.3 [0.05, 1.24]^m,q^	0.3 [0.05, 1.38]^m,q^	

^a^New calculated in this systematic review and meta-analysis

^b^Compared to patients with isolated esophageal atresia with or without tracheoesophageal fistula

^c^Compared to patients with the combined phenotype of esophageal atresia with or without tracheoesophageal fistula and anorectal malformation

^d^Compared to both, patients with isolated esophageal atresia with or without tracheoesophageal fistula and patients with the combined phenotype of esophageal atresia with or without tracheoesophageal fistula and anorectal malformation

^e^Folic acid supplementation in the first month of gestation

^f^Folic acid supplementation in the second month of gestation

^g^Daily total maternal intake of 5.13–7.79 mg

^h^Daily total maternal intake of 7.80–14.19 mg

^i^Daily total maternal intake of > 14.19 mg

^j^All ARM cases

^k^Isolated ARM cases only

^l^ARM with other defects

^m^Risk Ratio (RR)

^n^All cases with an anorectal malformation

^o^Anorectal malformation with no additional external anomalies

^p^Anorectal malformation with other caudal anomalies

^q^Anorectal malformation with multiple anomalies

**Table 3:** Associations between ARM and maternal use of asthma medication

		Maternal use of asthma medication			
Ref.	First author, year	Exposure	OR_crude_ [95% CI]	OR_adj_ [95% CI]	Adjustment/matching factors
[73]	Garne, 2015	Any anti-asthma medication	–	1.6 [1.08, 2.51]^b^	Registry and maternal age
			–	2.0 [1.30, 3.20]^c^	
		Beta-2-agonists in general	–	1.5 [0.84, 2.66]^b^	Center, maternal age and use of inhaled corticosteroids
			–	1.5 [0.79, 2.93]^c^	
		Inhaled beta-2-agonists	1.7 [1.08, 2.80]	1.5 [0.85, 2.68]^b^	Center, maternal age and use of corticosteroids
			2.3 [1.39, 3.75]	1.8 [0.92, 3.44]^c^	
			–	1.8 [1.11, 2.87]^b^	Center, maternal age
			–	2.1 [1.26, 3.54]^c^	
			–	1.5 [0.85, 2.69]^b^	Center, maternal age, systemic steroids and use of corticosteroids
			–	1.5 [0.79, 2.94]^c^	
			–	1.5 [0.83, 2.63]^b^	Center, maternal age, period (5-year intervals) and use of corticosteroids
			–	1.8 [0.92, 3.45]^c^	
			–	1.2 [0.35, 3.73]^b,f^	Center, maternal age, period (5-year intervals) and use of corticosteroids
			–	0.8 [0.23, 2.76]^c,f^	
		Inhaled corticosteroids	2.0 [1.10, 3.51]	1.5 [0.74, 3.02]^b^	Center, maternal age and use of beta-2-agonists
			3.3 [1.81, 5.98]	1.7 [0.73, 3.77]^c^	
			–	2.0 [1.09, 3.48]^b^	Center, maternal age
			–	2.8 [1.48, 5.17]^c^	
			–	1.5 [0.74, 3.03]^b^	Center, maternal age, systemic steroids and use of beta-2-agonists
			–	2.1 [0.94, 4.51]^c^	
			–	1.5 [0.75, 3.07]^b^	Center, maternal age, period (5-year intervals) and use of beta-2-agonists
			–	1.6 [0.72, 3.73]^c^	
			–	1.2 [0.31, 4.68]^b,f^	Center, maternal age, period (5-year intervals) and use of beta-2-agonists
			–	2.7 [0.78, 9.58]^c,f^	
			1.3 [0.41, 4.06]^a,b,f^	–	
		Combination treatments	–	1.0 [0.31, 3.24]^**b**^	Center, maternal age and use of short-acting beta-2-agonists
			–	1.4 [0.43, 4.28]^c^	
		Salbutamol	–	1.6 [0.87, 2.88]^b^	Center, maternal age and use of inhaled corticosteroids
			–	1.5 [0.78, 3.04]^c^	
[53]	Zwink and Choinitzki, 2015	Any anti-asthma medication	0.8 [0.02, 29.50]^d^	–	–
			0.3 [0.01, 16.63]^e^		
[67]	Yau, 2013	Pseudoephedrine	–	1.3 [0.8, 2.3]	Matched and adjusted for further factors (not specified)
[59]	Lin, 2012	Anti-inflammatory use	–	2.1 [1.09, 4.12]	Age, parity, race/ethnicity, education, alcohol use, smoking, gender, folic acid use, fever in first trimester
		Bronchodilator use	–	0.9 [0.38, 2.01]	
[42]	Källén and Otterblad Olausson, 2007	Any anti-asthma medication	–	1.7 [1.11, 2.56]	Year of birth, maternal age, parity, smoking, and number of previous miscarriages
		Inhaled corticosteroids	–	1.9 [1.00, 3.22]	
		Salbutamol	–	1.5 [0.50, 3.60^g^	
		Terbutaline	–	1.5 [0.82, 2.52]^g^	
		Salmeterol	–	2.5 [0.52, 7.37]^g^	
		Budesonide	–	1.9 [0.95, 3.42]^g^	

^a^New calculated in this systematic review and meta-analysis

^b^ARM vs. non-chromosomal anomaly control group

^c^ARM vs. chromosomal anomaly control group

^d^Compared to patients with isolated esophageal atresia with or without tracheoesophageal fistula

^e^Compared to patients with the combined phenotype of esophageal atresia with or without tracheoesophageal fistula and anorectal malformation

^f^Cases with no other multiple malformation only (isolated)

^g^Risk Ratio (RR)

**Table 4:** Associations between ARM and maternal use of thyroid medication

		Maternal use of thyroid medication			
Ref.	First author, year	Exposure	OR_crude_ [95% CI]	OR_adj_ [95% CI]	Adjustment/matching factors
[53]	Zwink and Choinitzki, 2015	Thyroid hormone supplementations	1.0 [0.25, 3.74]^b^	–	–
			0.4 [0.10, 1.54]^c^		
[75]	Källén and Wikner, 2014	Thyroxin	1.9 [1.00, 1.85]^a^	–	–
[61]	Browne, 2009	Anti-thyroid medication	8.6 [1.7, 40.2]	–	–

^a^Risk Ratio (RR)

^b^Compared to patients with isolated esophageal atresia with or without tracheoesophageal fistula

^c^Compared to patients with the combined phenotype of esophageal atresia with or without tracheoesophageal fistula and anorectal malformation

**Table 5:** Associations between ARM and maternal use of psychiatric drugs

		Maternal use of psychiatric drugs			
Ref.	First author, year	Exposure	OR_crude_ [95% CI]	OR_adj_ [95% CI]	Adjustment/matching factors
[52]	Furu, 2015	Any SSRI	1.5 [0.95, 2.37]	1.4 [0.88, 2.32]	Maternal age, year of birth, birth order, smoking, maternal diabetes, and country, and use of other prescribed drugs (antiepileptics, anxiolytics and hypnotics, and angiotensin converting enzyme inhibitors)
		Sertraline	2.8 [1.33, 5.92]	2.5 [1.09, 5.57]	
		Citalopram	1.6 [0.70, 3.48]	1.5 [0.64, 3.25]	
[50]	Wemakor, 2015	Any SSRI	–	2.5 [1.06, 5.68]	Registry
		Fluoxetine	–	2.6 [0.60, 10.91]	
		Paroxetine	–	2.8 [0.66, 11.96]	
		Citalopram	–	2.2 [0.29, 16.63]	
		Sertraline	–	–	
		Escitalopram	–	6.0 [0.73, 49.62]	
[76]	Källén, 2013	Opioids	–	–	–
		Anticonvulsants	2.9 [0.96, 6.86]^a^	–	
		Neuroleptics^b^	–	–	
		Dixyrazine or prochlorperazine	–	–	
		Sedatives or hypnotics	1.2 [0.33, 3.06]^a^	–	
		Antidepressants	1.2 [0.66, 2.10]	–	
[54]	Polen, 2013	Antidepressants^c^	–	–	–
		Venlafaxine^c^	–	–	
[56]	Reis and Källén, 2010	Antidepressants	1.1 [0.40, 2.36]^a^	–	–
[68]	Alwan, 2007	Any SSRI	1.0 [0.4, 2.0]	0.7 [0.3, 1.8]	Maternal race or ethnic group, presence or absence of maternal obesity, presence or absence of maternal smoking, and family income
[64]	Louik, 2007	Any SSRI	–	1.9 [0.8, 4.3]	Maternal age, maternal race or ethnic group (self-reported), maternal education, year of last menstrual period/study center (a composite variable), parity, first-trimester smoking status, first-trimester alcohol consumption, any family history of a birth defect, history of a cardiac defect in a first-degree relative, prepregnancy body-mass index, seizures, diabetes mellitus, hypertension, infertility, any use of folic acid, and first-trimester use of folic acid
		Fluoxetine	–	1.4 [0.3, 6.1]	
		Paroxetine	–	1.0 [0.1, 7.8]	
		Citalopram	–	3.0 [0.3, 28.2]	
		Sertraline	–	4.4 [1.2, 16.4]	
		Non-SSRI antidepressant	–	2.2 [0.6, 7.8]	
[65]	Czeizel, 2003	Diazepam in entire pregnancy Diazepam in second-third months of gestation	2.2 [1.2, 3.9]	1.9 [1.1, 3.3]	–
			10.0 [1.3, 78.1]	5.2 [1.4, 19.7]	
[43]	Bonnot, 2001	Lorazepam	–	6.2 [2.44, 15.74]	Maternal age and parity

*SSRI* selective serotonin reuptake inhibitors

^a^Risk Ratio (RR)

^b^Neuroleptics other than dixyrazine or prochloperazine

^c^Only one case was exposed to antidepressants

**Table 6:** Associations between ARM and maternal use of painkiller

		Maternal use of painkiller			
Ref.	First author, year	Exposure	OR_crude_ [95% CI]	OR_adj_ [95% CI]	Adjustment/matching factors
[58]	Hernandez, 2012	Aspirin	1.3 [0.79, 2.03]	–	–
		Ibuprofen Naproxen	1.1 [0.89, 1.42]	–	
			1.3 [0.84, 2.01]	–	
[60]	van Gelder, 2011	Non-steroidal anti-inflammatory drugs	1.4 [0.2, 10.7]	–	–
[71]	Czeizel, 2000	Acetylsalicylic acid	–	2.3 [0.9, 5.6]^b^	Adjusted for maternal age, birth order, acute and chronic maternal disorders and other drug use
				1.2 [0.7, 2.8]^c^	
				1.3 [0.5, 3.9]^d^	
[79]	Heinonen, 1977	Aspirin^a^	–	–	–

^a^The prevalence per 10,000 live births was 8.75

^b^A population control group, including maternal self-reported and medically documented drug use

^c^Medically documented drug use

^d^Patient control group

**Table 7:** Associations between ARM and maternal use of anti-infectives

		Maternal use of anti-infectives			
Ref.	First author, year	Exposure	OR_crude_ [95% CI]	OR_adj_ [95% CI]	Adjustment/matching factors
[63]	Crider, 2009	Any antibacterial medication use	–	1.0 [0.7, 1.3]	Maternal age, race, education, prepregnancy BMI, time from the estimated date of delivery to the interview, use of folic acid or multivitamins, and any periconceptional smoking or alcohol use
		Penicillins	–	0.8 [0.5, 1.2]	
		Erythromycins	–	1.0 [0.4, 2.1]	
		Nitrofurantoins	–	1.1 [0.4, 3.0]	
		Sulfonamides	–	1.0 [0.4, 2.9]	
		Cephalosporins	–	1.6 [0.7, 3.5]	
[62]	Carter, 2008	Antifungal drugs	–	1.4 [0.66, 3.06]	Pregnancy BMI, maternal education
[70]	Czeizel, 2001	Cephalexin	0.7 [0.1, 3.4]	–	–
		Cefuroxime	1.0 [0.0, 51.9]		
[72]	Stoll, 1997	Antibiotics	0.6 [0.23, 1.47]	–	–

**Table 8:** Associations between ARM and maternal use of drugs against nausea and vomiting

		Maternal use of drugs against nausea and vomiting			
Ref.	First author, year	Exposure	OR_crude_ [95% CI]	OR_adj_ [95% CI]	Adjustment/matching factors
[49]	Pasternak, 2013	Metoclopramide	0.7 [0.34, 1.54]^c^	0.8 [0.36, 1.66]^b^	Matched and adjusted for hospitalization for hyperemesis gravidarum or nausea and vomiting, and use of other antiemetics in the first trimester
[78]	Källén and Mottet, 2003	Meclozine	2.3 [0.99, 4.50]^a^	–	–

^a^Risk Ratio (RR)

^b^Prevalence odds ratio (POR)

^c^Neuroleptics other than dixyrazine or prochloperazine

**Table 9:** Associations between ARM and maternal use of sexual hormones

		Maternal use of sexual hormones			
Ref.	First author, year	Exposure	OR_crude_ [95% CI]	OR_adj_ [95% CI]	Adjustment/matching factors
[55]	Reefhuis, 2011	Clomiphene citrate	1.2 [0.6, 2.3]	1.2 [0.6, 2.3]	Maternal age, maternal race, parity, previous miscarriages, maternal education, smoking, alcohol use, obesity, and folic acid use
[72]	Stoll, 1997	Estrogens	0.1 [0.03, 0.63]	–	–

**Table 10:** Associations between ARM and maternal use of other medical drugs

		Maternal use of other medical drugs			
Ref.	First author, year	Exposure	OR_crude_ [95% CI]	OR_adj_ [95% CI]	Adjustment/matching factors
[72]	Stoll, 1997	All assessed medications^c^	0.04 [0.004, 0.32]^a^	–	–
			0.03 [0.003, 0.27]^b^		
		Antispasmodics Other miscellaneous medication	0.4 [0.16, 1.18]		
			0.5 [0.19, 1.32]		
[74]	Angerpointer, 1981	All assessed medications^d^	0.7 [0.38, 1.43]	–	–

^a^Cases with no other multiple malformation (isolated)

^b^Cases with other multiple malformations

^c^Included antibiotics, antispasmodics, estrogens and other miscellaneous medication

^d^Included antiemetic, analgetic, laxative and antihypotensive drugs, and iron preparations

### Dietary supplements

Ten studies reported on the association between maternal use of dietary supplements before or during pregnancy and infants born with an anorectal malformation, among them seven studies on folic acid, six studies on multivitamins, and each one study on vitamin E and iron intake (Table 2).

The study by Czeizel et al. [66] reported on a significantly protective association of folic acid supplementation with ARM when mothers used it in the second month of gestation (OR_crude_, 0.4; 95% CI, 0.17–0.88; *P* = 0.01). In contract, the use of folic acid in the first month of gestation was not significant (OR_crude_, 0.5; 95% CI, 0.17–1.23; *P* = 0.12). In the study by Myers et al. [51] different ARM groups were used. There was a significantly protective association with all ARM cases (RR_crude_, 0.5; 95% CI, 0.29–0.88) and a marginally protective association with ARM cases with no additional external anomalies (RR_crude_, 0.5; 95% CI, 0.24–1.04). After adjustment for maternal age the association with all ARM cases was weakened (RR_adj_, 0.6; 95% CI, 0.33–1.07) and the association with ARM cases with no additional external anomalies became insignificant (RR_adj_, 0.7; 95% CI, 0.31–1.42).

A marginally increased risk for multivitamins was only reported in the study by van Rooij et al. [27] (OR_crude_, 1.6; 95% CI, 1.0–2.7; *P* = 0.09). No other study could confirm an association with ARM. The study by Gilboa et al. [57] categorized maternal vitamin E intake during and before pregnancy into three classes (daily total intake of 5.13–7.79 mg, daily total intake of 7.80–14.19 mg, daily total intake of > 14.19 mg). A marginally increased risk was observed for the exposure group 7.80–14.19 mg per day only (OR_adj_, 1.7; 95% CI, 1.01–2.72). There was no association with maternal iron intake before or during pregnancy.

**Fig. 2:**
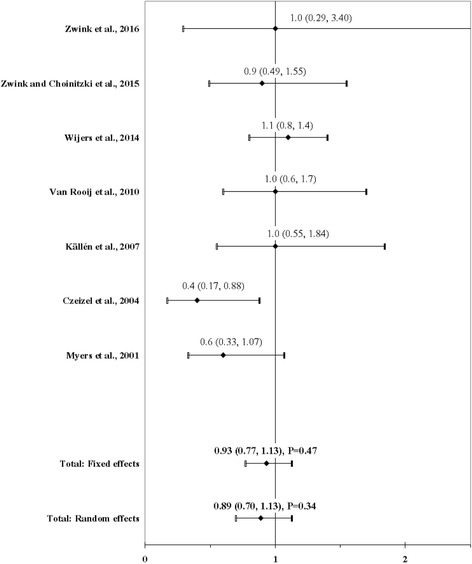
Forest plot for maternal use of folic acid

The result of the meta-analysis on the association between maternal use of folic acid and ARM infants is shown in Fig. 2. From the study by Zwink and Choinitzki et al. [53] we used the OR calculated with the group of control infants with esophageal atresia with or without tracheoesophageal fistula, from the study by Czeizel et al. [66] the OR calculated for supplementation in the second month of gestation as ARM is known to develop between the 5th–8th week of gestation, from the study by Wijers et al. [41] the OR calculated for all ARM cases, and from the study by Myers et al. [51] the RR calculated with all ARM cases. The I^2^ statistic indicated heterogeneity across studies (χ^2^ = 7.71; *P* = 0.26; I^2^ = 22.2%). The estimated heterogeneity variance was tau^2^ = 0.0226. In meta-analysis, no significant association was observed in pooled analyses using the fixed effects model (OR, 0.93; 95% CI, 0.77–1.13; *P* = 0.47). There was a weak evidence of publication bias (Kendall’s tau = − 1.65, *P* = 0.10; Egger’s t value = − 1.72, *P* = 0.15).

**Fig. 3:**
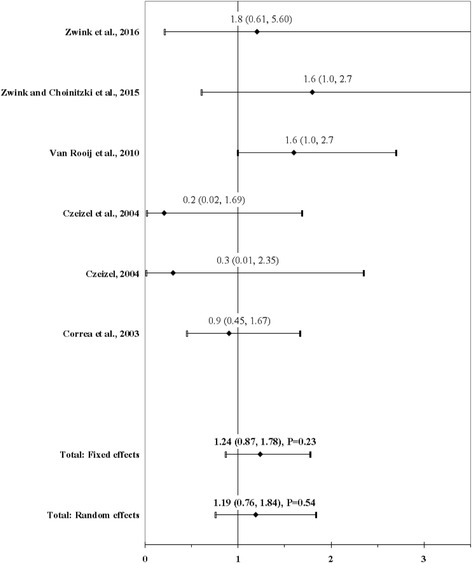
Forest plot for maternal use of multivitamins

The result of the meta-analysis on the association between maternal use of multivitamins and ARM infants is shown in Fig. 3. From the study by Zwink and Choinitzki et al. [53] we used the OR calculated with the group of control infants with esophageal atresia with or without tracheoesophageal fistula. The I^2^ statistic indicated low heterogeneity across studies (χ^2^ = 6; *P* = 0.31; I^2^ = 16.7%). The estimated heterogeneity variance was tau^2^ = 0.0523. No significant association was observed in pooled analyses using a fixed effects model (OR, 1.24; 95% CI, 0.87–1.78; *P* = 0.23). There was no evidence of publication bias (Kendall’s tau = − 1.32, *P* = 0.19; Egger’s t value = − 1.61, *P* = 0.18).

### Asthma medication

Five studies reported on the association between maternal use of asthma medication before or during pregnancy and infants born with an anorectal malformation (Table 3). The use of any anti-asthma medication showed a significant association with ARM in the studies by Källén and Otterblad Olausson [42] (OR_adj_, 1.7; 95% CI, 1.11–2.56) and Garne et al. [73]. In the latter study two different control groups were used. Both, the comparison of ARM with non-chromosomal anomalies (OR_adj_, 1.6; 95% CI, 1.08–2.51) and the comparison of ARM with chromosomal anomalies (OR_adj_, 2.0; 95% CI, 1.30–3.20) was significant for any anti-asthma medication. In contrast, the study by Zwink and Choinitzki et al. [53] could not find an association with ARM. The study by Lin et al. [59] observed an association between anti-inflammatory use and ARM (OR_adj_, 2.1; 95% CI, 1.09–4.12).

The use of inhaled corticosteroids showed a significant risk for ARM in the study by Garne et al. [73], independent of the used control group (ARM vs. non-chromosomal anomalies: OR_crude_, 2.0; 95% CI, 1.10–3.51; *P* = 0.04 and ARM vs. chromosomal anomalies: OR_crude_, 3.3; 95% CI, 1.81–5.98; *P* = 0.02). Both results remain significant after adjustment for center and maternal age (ARM vs. non-chromosomal anomalies: OR_adj_, 2.0; 95% CI, 1.09–3.48 and ARM vs. chromosomal anomalies: OR_adj_, 2.8; 95% CI, 1.48–5.17). However, results became insignificant after adjustment for more than those two covariates. The study by Källén and Otterblad Olausson [42] observed a marginally increased risk for inhaled corticosteroids (OR_adj_, 1.9; 95% CI, 1.00–3.22).

Beta-2-agonists in general were not associated with ARM, whereas inhaled beta-2-agonists showed a significant association with ARM in the study by Garne et al. [73] (ARM vs. non-chromosomal anomalies: OR_crude_, 1.7; 95% CI, 1.08–2.80; *P* = 0.03 and ARM vs. chromosomal anomalies: OR_crude_, 2.3; 95% CI, 1.39–3.75; *P* = 0.003). After adjustment for covariates the association with the non-chromosomal anomaly control group became insignificant, the association with the chromosomal anomaly control group was weakened (OR_adj_, 1.8; 95% CI, 0.92–3.44). A suggestive association with ARM was observed for the use of budesonide in the study by Källén and Otterblad Olausson [42] (OR_adj_, 1.9; 95% CI, 0.95–3.42).

**Fig. 4:**
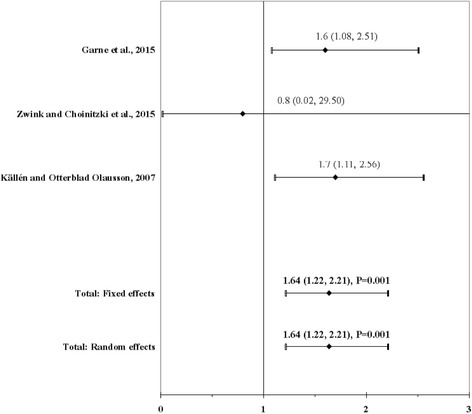
Forest plot for maternal use of any anti-asthma medication

The result of the meta-analysis on the association between maternal use of any anti-asthma medication and ARM infants is shown in Fig. 4. From the study by Garne et al. [73] we used the OR calculated with the non-chromosomal anomaly control group and from the study by Zwink and Choinitzki et al. [53] the OR calculated with the group of control infants with esophageal atresia with or without tracheoesophageal fistula. The I^2^ statistic indicated homogeneity across the three studies (χ^2^ = 0.19; *P* = 0.91; I^2^ = 0%). In meta-analysis, a significant association was found for maternal use of any anti-asthma medication before or during pregnancy using a fixed effects model (OR, 1.64; 95% CI, 1.22–2.21; *P* = 0.001). There was no evidence of publication bias (Kendall’s tau = − 1.57, *P* = 0.12; Egger’s t value = − 1.96, *P* = 0.30).

### Thyroid medication

Three studies reported on the association between maternal use of thyroid medication before or during pregnancy and infants born with an anorectal malformation (Table 4). The study by Browne et al. [61] reported on a significant association between anti-thyroid medication and ARM (OR_crude_, 8.6; 95% CI, 1.7–40.2; *P* = 0.005) and the study by Källén et al. [75] on a marginally increased risk for thyroxin intake (RR_crude_, 1.9; 95% CI, 1.00–1.85). In contrast, the study by Zwink and Choinitzki et al. [53] could not confirm the finding.

### Psychiatric drugs

Nine studies reported on the association between maternal use of psychiatric drugs before or during pregnancy and infants born with an anorectal malformation (Table 5). The study by Wemakor et al. [50] reported a significant association for any selective serotonin reuptake inhibitors (SSRI) (OR_adj_, 2.5; 95% CI, 1.06–5.68). In addition, the study by Furu et al. [52] observed a suggestive association with any SSRI (OR_crude_, 1.5; 95% CI, 0.95–2.37; *P* = 0.08). The result became insignificant after adjustment for covariates (OR_adj_, 1.4; 95% CI, 0.88–2.32). Neither the study by Alwan et al. [68] nor the study by Louik et al. [64] could confirm an association of any SSRI with ARM. Individual SSRI classes were assessed by Furu et al. [62] and Louik et al. [64]. Both studies reported on an increased risk for sertraline during and before pregnancy (Furu et al.: OR_adj_, 2.5; 95% CI, 1.09–5.57; Louik et al.: OR_adj_, 4.4; 95% CI, 1.2–16.4). Due to the small sample size, confidence intervals were very wide in both studies. However, there was no increased risk for citalopram.

Three studies assessed antidepressants in general [54, 56, 76]. None of them could find an association with ARM.

A marginally increased risk for diazepam was found in the study by Czeizel et al. [65] in both, in the entire pregnancy (OR_adj_, 1.9; 95% CI, 1.1–3.3) and in the second-third months of gestation (OR_adj_, 5.2; 95% CI, 1.4–19.7). The study by Bonnot et al. [43] showed a significant association between the use of lorazepam before or during pregnancy and ARM (OR_adj_, 6.2; 95% CI, 2.44–15.74). No association could be found in the study by Källén et al. [76] for other sedatives or hypnotics (RR_crude_, 1.2; 95% CI, 0.33–3.06). A suggestive association with ARM was observed for the use of anticonvulsants in the study by Källén et al. [76] (RR_crude_, 2.9; 95% CI, 0.96–6.86).

**Fig. 5:**
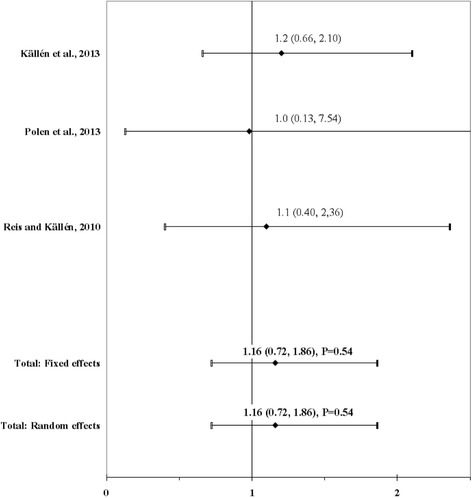
Forest plot for maternal use of antidepressants

The result of the meta-analysis on the association between maternal use of antidepressants and ARM infants is shown in Fig. 5. The I^2^ statistic indicated homogeneity across the three studies (χ^2^ = 0.05; *P* = 0.97; I^2^ = 0%). No significant association was found for maternal use of antidepressants before or during pregnancy using a fixed effects model (OR, 1.16; 95% CI, 0.72–1.86; *P* = 0.54). There was no evidence of publication bias (Kendall’s tau = − 1.57, *P* = 0.12; Egger’s t value = − 2.73, *P* = 0.22).

**Fig. 6:**
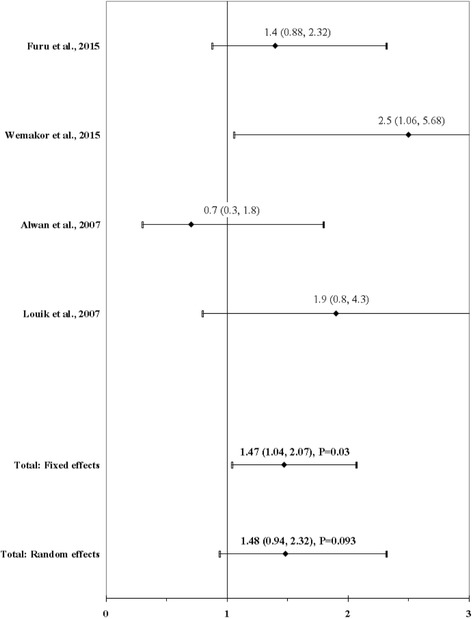
Forest plot for maternal use of any selective serotonin reuptake inhibitors (SSRI)

The result of the meta-analysis on the association between maternal use of any SSRI and ARM infants is shown in Fig. 6. The I^2^ statistic indicated heterogeneity across studies (χ^2^ = 4.57; *P* = 0.2061; I^2^ = 34%). The estimated heterogeneity variance was tau^2^ = 0.0744. No significant association was observed in pooled analyses using the random effects model (OR, 1.48; 95% CI, 0.94–2.32; *P* = 0.093). There was no evidence of publication bias (Kendall’s tau = − 0.68, *P* = 0.50; Egger’s t value = 0.05, P = 0.97).

**Fig. 7:**
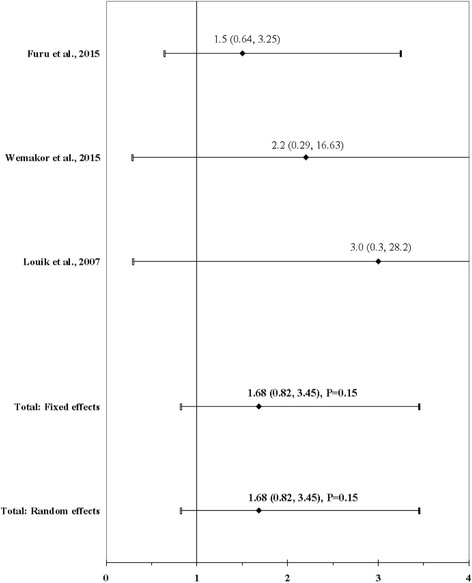
Forest plot for maternal use of citalopram

The result of the meta-analysis on the association between maternal use of citalopram and ARM infants is shown in Fig. 7. The I^2^ statistic indicated homogeneity across the three studies (χ^2^ = 0.39; *P* = 0.82; I^2^ = 0%). No significant association was found for maternal use of citalopram before or during pregnancy using a fixed effects model (OR, 1.68; 95% CI, 0.82–3.45; *P* = 0.15). There was no evidence of publication bias (Kendall’s tau = 1.57, *P* = 0.12; Egger’s t value = 4.48, *P* = 0.14).

**Fig. 8:**
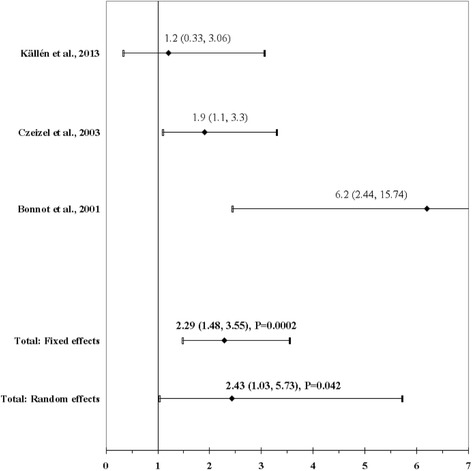
Forest plot for maternal use of hypnotics and benzodiazepine

The result of the meta-analysis on the association between maternal use of hypnotics and benzodiazepine and ARM infants is shown in Fig. 8. The I^2^ statistic indicated heterogeneity across studies (χ^2^ = 6.13; *P* = 0.047; I^2^ = 67.4%). The estimated heterogeneity variance was tau^2^ = 0.3820. In meta-analysis, a weak association was observed in pooled analyses using the random effects model (OR, 2.43; 95% CI, 1.03–5.73; *P* = 0.042). There was no evidence of publication bias (Kendall’s tau = − 0.52, *P* = 0.60; Egger’s t value = 0.22, *P* = 0.86).

### Painkiller

Four studies reported on the association between maternal use of painkillers before or during pregnancy and infants born with an anorectal malformation (Table 6). Only the study by Czeizel et al. [71] found a suggestive association between acetylsalicylic acid and ARM (OR_adj_, 2.3; 95% CI, 0.9–5.6) when using a population control group, including maternal self-reported and medical documented drug use.

### Anti-infectives

Among the four studies that reported on the association between maternal use of anti-infectives before or during pregnancy and infants born with an anorectal malformation (Table 7), none could find an association with ARM.

### Drugs against nausea and vomiting

Two studies reported on the association between maternal use of drugs against nausea and vomiting before or during pregnancy and infants born with an anorectal malformation (Table 8). The study by Källén and Mottet [78] found a marginally increased risk for meclozine intake (RR, 2.3; 95% CI, 0.99–4.50). There was no association with metoclopramide and ARM (POR_adj_, 0.8; 95% CI, 0.36–1.66) [49].

### Sexual hormones

Among the two studies that reported on the association between maternal use of sexual hormones before or during pregnancy and infants born with an anorectal malformation (Table 9), neither the study by Reefhuis et al. [55] not the study by Stoll et al. [72] could find an association for clomiphene citrate (OR_adj_, 1.2; 95% CI, 0.6–2.3) or estrogens (OR, 0.1; 95% CI, 0.03–0.63).

### Other medical drugs

Among the two studies that reported on the association between maternal use of other medical drugs, among them antispasmodics, other miscellaneous medication and mixed preparations, before or during pregnancy and infants born with an anorectal malformation (Table 10), none could find an association with ARM.

## Discussion

This systematic review and meta-analysis summarized the results of 37 epidemiological studies on the association between maternal medical drug intake and infants born with an anorectal malformation reported between 1977 and April 2017. The majority of the studies were conducted in the United States. Case numbers ranged from six ARM cases in the study by Bonnot et al. [43] to 799 ARM cases in the study by Furu et al. [52]. Studies were also heterogeneous with respect to period ingestion of medical drug use, control selection and adjustment for covariates. Less than half of the studies classified the administrated medical drug either by the international ATC classification or linked medication to the Slone Drug Dictionary [50, 52, 54, 56–59, 61–63, 67, 68, 73, 75, 76, 78]. Meta-analysis was done for medical drugs reported on in at least three studies, i.e. maternal use of folic acid, multivitamins, any anti-asthma medication, any selective serotonin reuptake inhibitors (SSRI), antidepressants, citalopram,, and hypnotics and benzodiazepine.

There is a great discrepancy in the reported results on the association between the different maternal medical drugs and ARM which impede comparability. As the exact active agent, dose and frequency of medical drug use is not reported in all studies, one can only speculate about possible detrimental effects on embryogenesis due to high dose/overdose of medical drugs. Such effects have been already observed in previous studies with vitamin A and etretinate for other birth defects [39, 83]. On the other hand, a vitamin A deficiency during pregnancy may also lead to birth defects like ARM [40]. To our knowledge, no previous epidemiological study exists assessing the association between vitamin A and ARM. However, six studies assessed multivitamins in general, resulting in no association with ARM in meta-analysis.

In contrast, the use of folic acid before and during pregnancy is discussed as having a protective effect for birth defects, among them neutral tube defects, cleft lip and cleft palate and heart defects [84–86]. The German Society for Nutrition (DGE) [87] recommends a daily folic acid intake of 400 micrograms per day at least 4 weeks before conception and later 450–550 micrograms per day for all pregnant women. Unwanted side effects with the intake of folic acid are not known. Our meta-analysis, however, could not show any association with ARM. In contrast, the study by Faria et al. [88] observed in their experimental model a reducing effect in the incidence of ARM when inducing ethylenethiourea in rats.

Depressions are common in pregnancy. Previous studies have been shown that approximately 10–15% of all women suffer from peripartum depression [89]. It is suggested that depression during pregnancy might be a risk factor for preterm birth and small-for-gestational-age, and possibly also for low birthweight [90, 91]. Depending on the strength of the symptoms, depression can be either treated with psychotherapy, medical drug intake or in case of severe depression with clinical stay. Antidepressant use in first trimester pregnancy has been estimated at 1–8% [92–94]. The most frequently used antidepressants are selective serotonin reuptake inhibitors (SSRIs). Up to 2005, SSRIs were regarded to be safe in pregnancy. Thereafter, its safety was questioned as different studies reported on an association between congenital malformations and SSRI use in first trimester of pregnancy [52, 56, 95–97]. A consistently risk, however, could only be observed for congenital heart defects, including septal heart defects [98]. There are assumptions that the risk further strongly increases when using several SSRI before and during pregnancy [97]. With respect to interactions with other drugs, citalopram, escitalopram and sertraline are assumed to be better than other SSRIs [99]. In Germany, citalopram is the most common prescribe medical drug, followed by sertraline and escitalopram [100]. In this meta-analysis, no association was found for antidepressants or any SSRIs with ARM. Hypnotics and benzodiazepine, including diazepam, larozepam and sedatives or hypnotics, showed a more than doubled risk for ARM.

The prevalence of asthma in pregnancy is estimated to be 4–12% [101, 102]. Mothers with this chronic disease are recommended to continue their medication during pregnancy. Its medical treatment includes the use of beta-2-agonists for symptom relief (rescue treatment) and/or anti-inflammatory medications for reducing and preventing chronic inflammation in the airways. In literature, an increased risk with specific birth defects, among them malformations of the nervous system, respiratory system, and digestive system, esophageal atresia, omphalocele, cardiac defects, facial clefts and gastroschisis, could be observed [59, 73, 103]. However, it remains unclear whether the medication increases the risk for congenital malformations or the disease itself. In this meta-analysis, we could also confirm an increased risk for ARM. The risk was almost doubled in mothers who used any anti-asthma medication before and during pregnancy.

Disorders of thyroid function are divided into hypothyroidism with an extremely low level of metabolism hormone thyroxine, and hyperthyroidism with an excessive production of thyroid hormones. In general, changes in thyroid function are available in up to 15% of all pregnancies, with a prevalence of overt and subclinical hypothyroidism of approximately 0.4% and 2–3%, respectively, and of overt and subclinical hyperthyroidism of approximately 0.1–0.4% and 2%, respectively [104, 105]. An untreated hypo- or hyperthyroidism seems to increase the risk for early or stillbirths and underweight babies [106]. With a physician well-controlled medication intake, however, no adverse effects on the unborn child could be verified. Furthermore, only few studies reported on an association between thyroid medication and congenital malformations, with almost inconsistent results [79, 107–110]. The findings suggest an association for heart defects, central nervous system defects, intestinal defects, hypospadias, and limb deficiencies. For ARM, available results were contradictory and did not allow any conclusion.

When available, data on ARM infants with isolated anomalies (no additional major defects) were preferred in this review to data on ARM infants with multiple defects. Only three of the 37 reviewed studies looked at both groups [41, 59, 73]. Analyses, however, showed nearly the same results. Further six studies reported on isolated ARM cases only [53, 57, 65, 69–71]. The three studies by Wikner et al. [111], Yuskiv et al. [112] and Mastroiacovo et al. [113] were excluded because they analyzed ARM in a group with other major congenital malformations that could be not clearly differentiated and individually assigned to medical exposure, and which might thereby mix or dilute potential effects in case of diverse etiologies. The excluded studies did not find an association with the examined risk factors (multivitamin and vitamin A, respectively).

Looking at some other gastrointestinal malformations, asthma medication use during pregnancy also seem to increase the risk for esophageal atresia, omphalocele and gastroschisis [59, 73]. There is a suggestive association between any antibiotics and small intestinal atresia/stenosis as well as between any antibiotics and gastroschisis [63]. In addition, the use of SSRIs in pregnancy is also reported to be a risk for omphalocele and gastroschisis [50, 52, 54, 64, 68]. The use of vasoactive medication, including pseudoephedrine, acetaminophen, phenylpropanolamine, aspirin, ibuprofen, and acetaminophen, was reported to be an elevated risk for gastroschisis and small intestinal atresia [67, 114–118]. Furthermore, the use of anticonvulsants as well as a daily intake of vitamin E of more than 7.8 mg might increase the risk for small intestinal atresia/stenosis [57, 76].

The significant associations with ARM and some other gastro-intestinal malformations show clearly the need of a physician well-controlled medication intake during pregnancy to early detect a possible overdose or incorrect intake or even interactions due to multiple medication intake.

Our review has a number of limitations mostly resulting from the overall scarcity of published evidence. First, our meta-analysis was limited by the data provided in the individual studies. Not all studies provided risk estimates adjusted for potentially influential confounders, such as maternal age, periconceptional smoking, pregnancy BMI, race/ethnicity, education and parity. A homogenous epidemiological study is almost impossible to get. It is essential to adjust for potentially confounders, including interaction between various drugs. Doing not so makes a direct comparison of the results difficult and thus the interpretation in meta-analyses. Furthermore, so called “ad hoc” studies would be desirable. However, in studies assessing rare diseases, high efforts and costs are needed to achieve a suitable sample size. Due to the small number of studies, we decided to pool adjusted and crude values for meta-analyses. Second, the used medical drug was almost not exactly described in the studies, including medication classification with its active agents, dose and frequency of medical drug use, and the intake of multiple medications simultaneously and thereby possible occurring interactions. Third, studies did not differentiate between ARM phenotypes ranking from lower to higher forms with different genetic background [18]. Fourth, some studies used affected (malformed) control groups. Other studies used mixed controls of live-born malformed and healthy babies. A potential advantage of using malformed controls is potential reduction of response bias or recall bias that may occur when a non-malformed control group is used. On the other hand, observed associations may be biased if the risk factors of interest are also associated with the malformations of controls. Fifth, most sample sizes were small, so the power to detect associations was low. Sixth, despite the lack of indication of major publication bias, it is impossible to be ruled out completely, especially in the light of the low number of studies. Seventh, although we searched in three databases (PubMed, ISI Web of Knowledge and Scopus) and completed our search by reviewing related and cross-referencing literature, existence of relevant missing or up to now unpublished studies cannot be excluded. In addition, we had no contact to authors to help ensure all relevant studies were included. However, as part of the international network on anorectal malformations (ARM-Net), we were in regular contact with its experts. Finally, the restriction to English-language articles might also have an influence to the limited evidence for ARM. Nevertheless, non-English-language articles are also not all available on databases such as PubMed, ISI Web of Knowledge and Scopus.

## Conclusions

To our knowledge, our article is the first systematic review and meta-analysis that provides an overview of the available epidemiological studies that reported on the association between maternal medical drug use before and during pregnancy and ARM. Adequate evidence is still very limited, especially in regard to gene interaction. Separate report of isolated ARM and those cases with multiple defects should become standard. Due to small sample sizes, it is understandable that data are very often analyzed together. Approximately 64% of all ARM patients have one or more additional extra-anal anomalies and only 36% have an isolated ARM (no further major birth defect) [5]. Nevertheless, results may be biased if the potential risk factor of interest is associated with an additional extra-anal anomaly, such as kidney, renal or heart defect. To facilitate drug comparison and obtain meaningful results, international classifications such as the World Health Organization’s Anatomical Therapeutic Chemical Classification System with Defined Daily Doses (ATC/DDD) [80, 81], are required to specify exactly medical drugs as well as to prescribe its dose and frequency. In addition, the intake of multiple medications simultaneously and thereby possible occurring interactions must be considered. Furthermore, it may be an inherent bias source to distinguish between parental chronic disease and their drug treatment. For example, in a previous study [28], we found maternal respiratory disease as a periconceptional risk factor, but it remains unclear whether the disease or the medication (agents or additives) treatment represents the risk factor. The same was observed in the study by Acs et al. [119] with maternal dyspepsia. Thus, further symptoms have been to be investigated. Further multicenter or register-based studies are needed to clarify the role of maternal medical drug intake for the development of ARM.

## Abbreviations

ARM: Anorectal malformations
ATC/DDD: The World Health Organization’s Anatomical Therapeutic Chemical classification system with Defined Daily Doses
CI: Confidence interval
DGE: The German Society for Nutrition
OR: Odds ratio
RR: Risk ratio
SSRI: Selective serotonin reuptake inhibitors
USA: United States of America
